# Association of tear matrix metalloproteinase 9 immunoassay with signs and symptoms of dry eye disease: A cross-sectional study using qualitative, semiquantitative, and quantitative strategies

**DOI:** 10.1371/journal.pone.0258203

**Published:** 2021-10-18

**Authors:** You Hyun Lee, Seung-Pil Bang, Kyu-Young Shim, Myung-Jin Son, Harim Kim, Jong Hwa Jun

**Affiliations:** 1 Department of Ophthalmology, Keimyung University School of Medicine, Daegu, Republic of Korea; 2 Department of Biomedical Engineering, University of Rochester, Rochester, New York, United States of America; Save Sight Institute, AUSTRALIA

## Abstract

**Purpose:**

This study aimed to analyze the association of tear matrix metalloproteinase 9 (MMP-9) immunoassay with the severity of dry eye (DE) signs and symptoms through qualitative, semiquantitative, and quantitative evaluations of immunoassay band.

**Materials and methods:**

This cross-sectional study enrolled 320 eyes of 320 patients. The clinical signs of DE were assessed using the Ocular Surface Disorder Index (OSDI) score, visual analogue scale (VAS), tear breakup time (tBUT), tear volume evaluation by tear meniscometry, and staining scores of the cornea and conjunctiva by the Oxford grading scheme. The tear MMP-9 immunoassay results were interpreted using qualitative (positive or negative), semi-quantitative (reagent band density on a four-point scale: 0 = negative; 1 = weakly positive; 2 = moderately positive; 3 = strongly positive), and quantitative (ratio of reagent band density to control band density) indicators.

**Results:**

Positive MMP-9 immunoassay results were significantly related to shorter tBUT, tBUT ≤3 seconds, higher corneal staining score, corneal staining score ≥2, and conjunctival staining score ≥2. The semi-quantitative results of the MMP-9 immunoassay were positively correlated with higher corneal staining score (*r* = 0.122, *p* = 0.029) and negatively correlated with tBUT (*r* = -0.125, *p* = 0.025). However, in the quantitative analysis, none of the DE signs or symptoms were correlated to the band density of the MMP-9 immunoassay.

**Conclusions:**

The positive MMP-9 immunoassay results were related to the severity of ocular signs of DE. However, using quantitative measures of the MMP-9 immunoassay to assess the clinical severity of DE requires further investigation.

## Introduction

Dry eye (DE) is a multifactorial disease of the tears and ocular surface that results in visual disturbance and tear film instability, and it negatively impacts daily living, emotional well-being, and the ability to work [1, 2]. Typically, to assess the clinical severity of DE, patients’ subjective symptoms are evaluated using different types of dry eye syndrome questionnaires such as the National Eye Institute Visual Function Questionnaire-25, Ocular Surface Disease Index (OSDI), Standard Patient Evaluation of Eye Dryness Questionnaire, etc. [3–5]. Further, the clinical signs are usually assessed using the Schirmer test, tear breakup time (tBUT), fluorescein or lissamine green staining score of the cornea and conjunctiva, and evaluation of the meibomian gland [6, 7]. Recently, inflammation has been reported as one of the core mechanisms involved in the development of DE [8]. Considering these newly identified core mechanisms, tear hyperosmolarity and elevation of tear matrix metalloproteinase 9 (MMP-9) were added as diagnostic measures of DE: tear osmolarity > 300 mOsm/l or an inter-eye difference > 8 mOsm/l are considered as loss of homeostasis, which can be detected using the TearLab osmometer (San Diego, CA, USA) [9, 10]. MMP-9 is secreted from the ocular surface epithelium and its concentration in tears is normally below 40 ng/ml [11]. The inflammatory processes of DE induce the release of MMP-9, elevating its concentration in tears [12]. The InflammaDry (Quidel Corporation, San Diego, CA, USA) is a newly developed diagnostic tool that can detect MMP-9 in tears at a concentration > 40 ng/ml [13–15].

Various studies were performed to validate this new point-of-care MMP-9 immunoassay. Such studies mostly focused on the comparison of qualitative test results with the clinical symptoms and signs of DE. Messmer et al. [16] reported that decreased tBUT, severe meibomian gland dysfunction (MGD), ocular surface staining, and low Schirmer test results were significantly correlated with positive MMP-9 results. Chotikavanich et al. [17] mentioned that MMP-9 positivity is significantly correlated with the symptom severity scores, topographic surface regularity index, conjunctival and corneal fluorescein staining scores, and tBUT.

A previous study reported that MMP-9 concentration was correlated with tear osmolarity and Schirmer strip volume [18]. Park et al. [19] also demonstrated a good correlation between DE symptoms and semi-quantitative MMP-9 grading. Further, our previous study showed that the band density of InflammaDry increased proportionally with MMP-9 concentration in experimental conditions [20]. Therefore, we wanted to investigate whether strong band density is related to the severity of DE signs and symptoms. Furthermore, to the best of our knowledge, studies on the relationship between quantitative MMP-9 immunoassay test results and the clinical severity of DE are lacking. Therefore, this study determined the correlation between MMP-9 immunoassay results, interpreted using qualitative (positive or negative), semi-quantitative (reagent band density on a four-point scale: 0 = negative; 1 = weakly positive; 2 = moderately positive; 3 = strongly positive), and quantitative (ratio of the reagent band density to the control band density) indicators, and the clinical signs and symptoms of DE.

## Materials and methods

### Study design, and setting

This cross-sectional study enrolled 320 patients who visited our ophthalmic department from April 1, 2017, through October 1, 2018. The study adhered to the tenets of the Declaration of Helsinki and was approved by the institutional review board of Keimyung University Dongsan Hospital (IRB no. 2017-06-008). The patients signed informed consent for the use of their data. The study investigator collected clinical data and MMP-9 test results from the right eye of each enrolled patient.

### Clinical assessment of DE and data collected

#### Enrolled criteria

Patients with chief complaints of DE symptoms such as stinging, burning, and/or scratchy sensation in eyes and met at least one of the following four criteria were recruited: OSDI score >20, tBUT <5 seconds, tear meniscometry test results without anesthesia <5 mm/5 seconds, and corneal fluorescein staining results ≥1. Using these criteria, we tried to exclude as many mild DE patients as possible. Clinical evaluations were performed in the following sequence: OSDI questionnaire/visual analogue scale (VAS) score, tear meniscometry, tear MMP-9 immunoassay, tBUT, corneal and conjunctival staining scores, and meibomian gland evaluation after topical proparacaine (Paracaine; Hanmi Pharm, Seoul, South Korea) instillation. Patients with active ocular infection, who were pregnant, or lacrimal drainage disorders such as lacrimal punctal stenosis, deformed lacrimal punctum, canalicular anomalies, and nasolacrimal duct obstruction; those receiving topical or systemic corticosteroid treatment or immunomodulatory therapy within 1 month; those who had fluorescein allergy, cornstarch or dacron allergy, undergone ocular surgery within 6 months, or had ocular trauma in the previous 3 months; and those who wore contact lenses within 72 hours were excluded.

#### Subjective symptoms

Patients underwent full ophthalmologic examination. Subjective symptoms were measured using the OSDI questionnaire [4]. OSDI scores range from 0 to 100, where 0 indicates no disability and 100 indicates complete disability. The degree of ocular pain was documented using the VAS, where 0 indicates no pain and 10 indicates worst possible pain [21].

#### Tear strip meniscometry for tear volume evaluation

The tear volume was measured by tear meniscometry (SMTube^®^; Echo Electricity Co., Ltd., Fukushima, Japan). After blinking voluntarily 2 to 3 times, the tip of the tear meniscometry tube was applied to the lateral third area of the lower lid tear lake for 5 seconds. Tear volume was recorded by the length (in millimeters) of the stained tear column.

#### Tear MMP-9 point-of-care test

**Fig 1:**
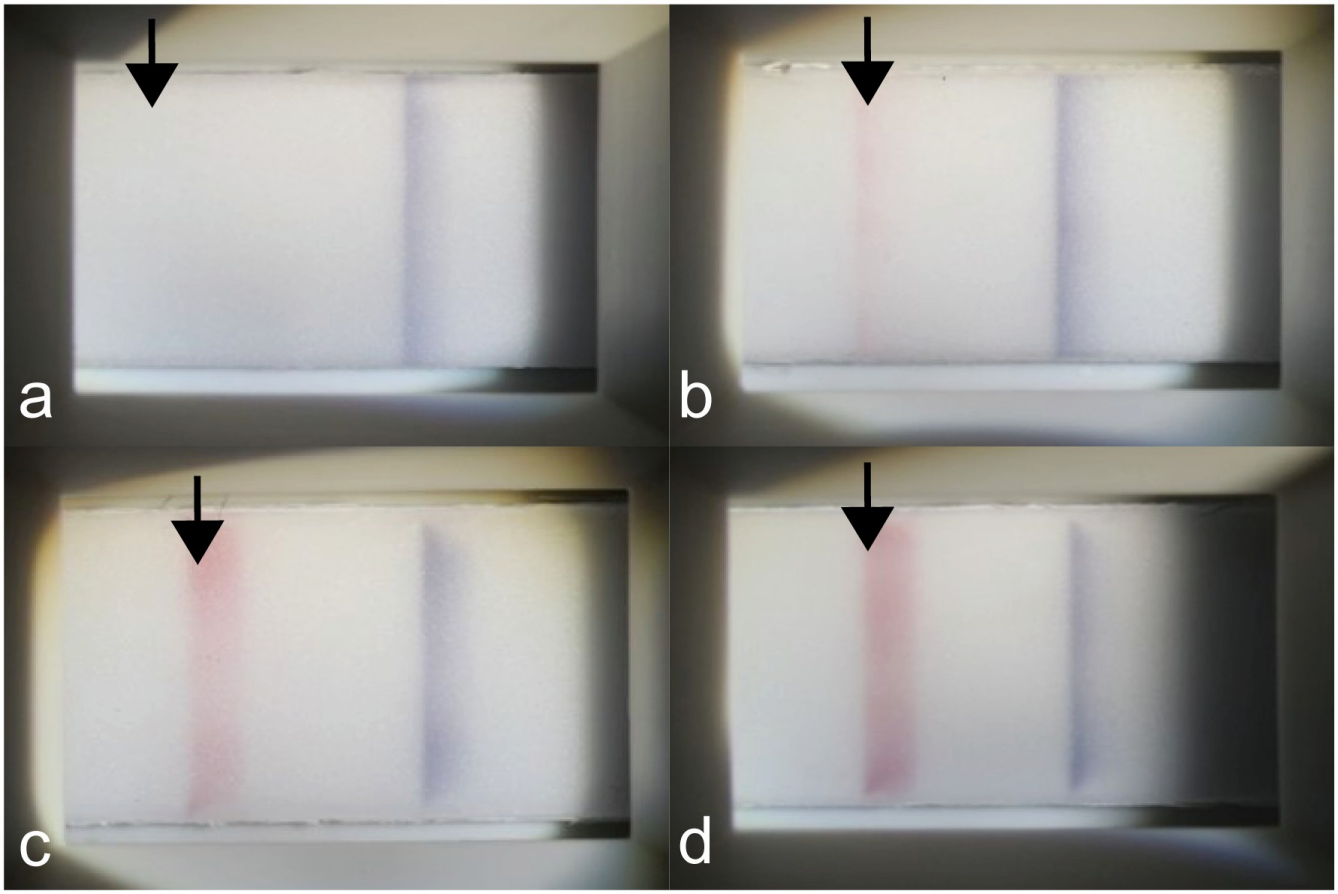
Semiquantitative analysis of matrix metalloproteinase 9 immunoassay according to the colour intensity of reagent band (arrow). (a) Negative. (b) Weak positive. (c) Moderate positive. (d) Strong positive.

**Fig 2:**
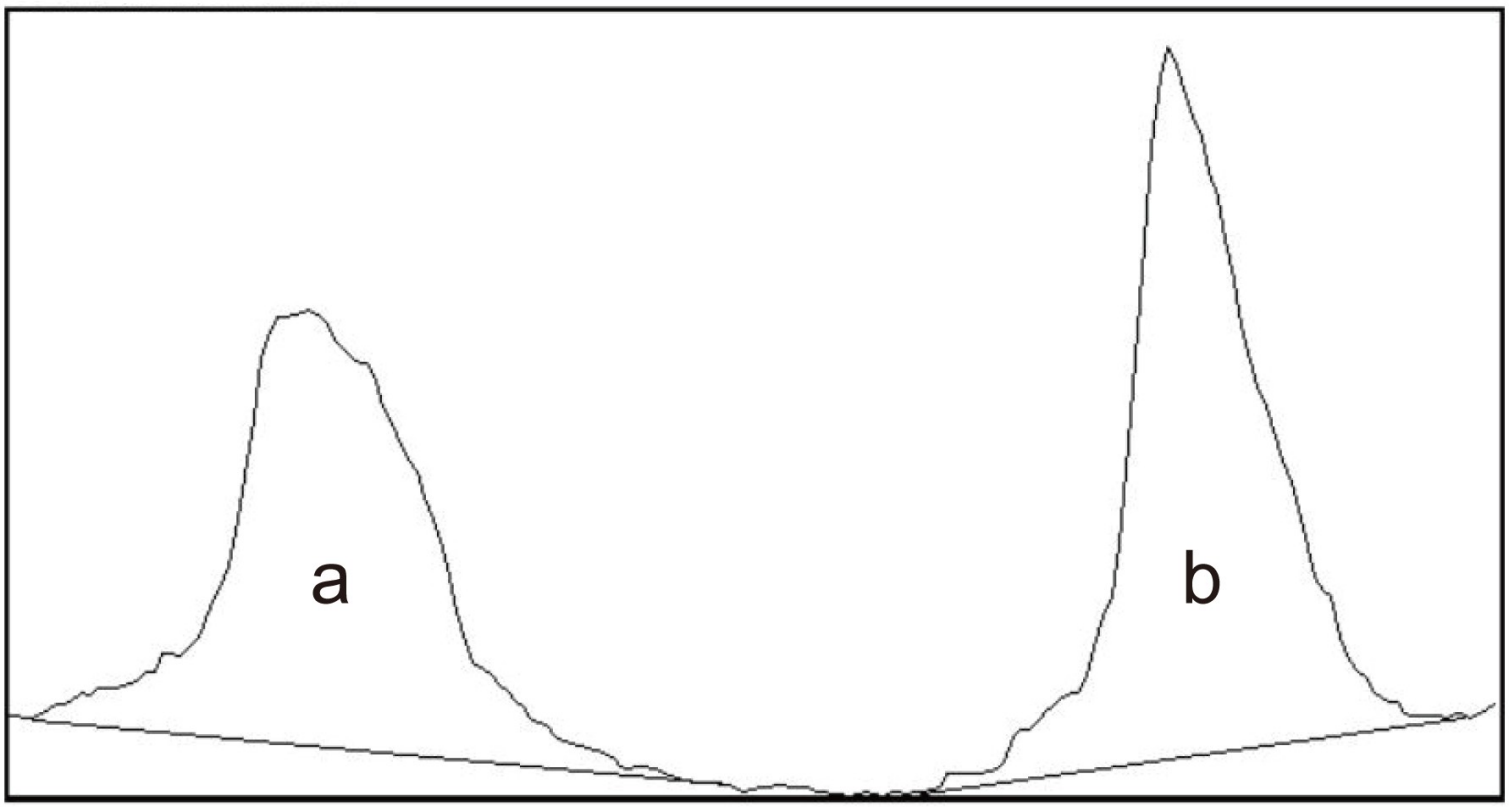
Quantitative analysis of matrix metalloproteinase 9 immunoassay using Image J software version 1.44p (https://imagej.nih.gov/ij/). (a) Reagent band density area. (b) Control band density area.

The tear MMP-9 immunoassay (InflammaDry) test was performed according to the manufacturer’s instruction by a single examiner (JHJ). To collect a tear sample, the sample collector was dabbed three times in three different locations of the inferior conjunctival palpebrae (temporal, middle, nasal; from nasal to temporal direction) and was placed against the temporal inferior palpebral conjunctiva for an additional 5 seconds. After that, the sample collector was snapped to a test cassette. After 5 seconds, the absorbent tip was immersed into a buffer solution. To evaluate the result band density of the test line under the same conditions, a photograph of the result window was taken with a slit-lamp biomicroscope mounted with a single-lens reflex camera (Canon EOS 700D, setting: ISO 400, shutter speed 1/200 sec; Canon USA, Melville, NY, USA) 20 minutes after the test initiation. Test results were interpreted using qualitative, semiquantitative, and quantitative. Qualitative analysis was performed using a two-point scale: 0 = negative, 1 = positive). Results were considered assay positive when there were other bands aside from than the faint band. Semiquantitative analysis based on the colour intensity of the test (red) line was performed by a single clinician (JHJ) using a four-point scale: 0 = negative; 1 = weak positive; 2 = moderate positive; 3 = strong positive (Fig 1). Quantitative analysis of red line band density was measured with ImageJ version 1.44p (National Institutes of Health, Bethesda, MD, USA) by three different experimenters (YHL, M-JS, and HK) [22]. First, the image of the test result was mounted to the ImageJ software and converted to 8-bit colour. Second, a square that included both the reagent and control bands was drawn. Third, the first lane was selected, followed by the select plot lanes in the analyze tool. Fourth, two peak points were checked, and two lines that constituted the most ideal parabola were drawn. Lastly, the wand tool was select, and the area that represents the reagent (Fig 2a) and control band (Fig 2b) densities was clicked. The quantitative results were calculated by the ratio of the reagent band density to the control band density from ImageJ. The clinicians who interpreted these results were blinded to the patients’ clinical characteristics.

#### tBUT

The tBUT was assessed by a wetted fluorescein strip (Haag-Streit AG, Koeniz, Switzerland) touched into the lower inferotemporal bulbar conjunctiva. Patients were instructed to blink, and the interval time between the last blink and the first appearance of dark spots in the tear film was recorded using a stopwatch under blue-light illumination with a biomicroscope and x10 magnification.

#### Corneal and conjunctival stain scores

Corneal and conjunctival staining was conducted using fluorescein instillation into the tear film, and the score was measured using the Oxford grading scheme. The degree of staining was based on the number of dots on a series of panels (A–E); the staining score ranges from 0 to 5 for each panel, for a total possible score ranging from 0 to 15 for the exposed interpalpebral conjunctiva and cornea [23]. The conjunctival stain score was measured for the nasal and temporal sides of the right eye, and the total score was used for the correlation analysis.

#### Meibomian gland evaluation

MGD was assessed according to its secretion turbidity and expressibility. Turbidity was graded from 0 to 3: clear = 0; cloudy = 1; granular = 2; inspissated = 3. Expressibility was assessed after application of topical anesthesia and graded from 0 to 3: 0 = clear meibum with easily expressed, 1 = cloudy meibum expressed with mild pressure, 2 = thick cloudy meibum expressed with more than moderate pressure; 3 = meibum not expressed, even with hard pressure [24, 25].

#### Evaluation of clinical severity of DE

The clinical severity of DE was assessed by comparing the DE signs and symptoms across different categories (MMP-9 positive versus negative, using the qualitative indicator; MMP-9 negative versus weakly positive versus moderately positive versus strongly positive, using the quantitative indicator) of different MMP-9 immunoassay indicators. An indicator category was considered as relatively more severe if the patient group showed worse DE signs and symptoms.

#### Statistical methods

Data were calculated as mean ± standard deviation (SD), unless otherwise specified. Statistics were analyzed using SPSS version 12.0 (IBM, Chicago, IL, USA). The between-group differences in age, OSDI score, VAS score, tear meniscometry, tBUT, corneal staining, conjunctival staining, turbidity, and expressibility of the meibomian gland were compared using independent t-test. The relationship of various systemic diseases related to the DE and MMP-9 positivity was analyzed using chi-square test. The Spearman correlation test was performed between semiquantitative MMP-9 results and age, OSDI score, VAS score, tear meniscometry results, tBUT, corneal staining score, conjunctival staining score, and MGD score. The Pearson correlation test was performed between quantitative results of MMP-9 and other age, OSDI score, VAS score, tear meniscometry results, tBUT, corneal staining score, conjunctival staining score, and MGD score. Two-sided p values <0.05 were considered statistically significant.

## Results

### Qualitative results of the MMP-9 immunoassay

#### Demographics of the study populations

A total of 320 patients (320 eyes) were included in the study. The mean age was 58 ± 13 years (range, 14–90 years), and 97 (30%) were men. The demographic data of patients according to MMP-9 positivity are shown in Table 1. There are no significant differences between the groups, and the presence of systemic autoimmune disease (rheumatoid arthritis, scleroderma, systemic lupus erythematosus, overlap syndrome, or secondary Sjögren disease) and Sjögren disease were not significantly different (p>0.05).

**Table 1:** Demographics in MMP-9 positive versus negative patients.

Parameters	MMP-9 positive (n = 205)	MMP-9 negative (n = 115)	*P* value
Age, mean (SD)	59 (14)	58 (13)	0.484
Gender, n (%) male	65 (31.7%)	32 (27.8%)	0.527
Systemic autoimmune disease (except Sjögren syndrome), n (%)	20 (9.8%)	14 (12.2%)	0.571
Sjögren syndrome, n (%)	26 (12.7%)	9 (7.8%)	0.197
Diabetes, n (%)	19 (9.3%)	5 (4.3%)	0.125
Hypertension, n (%)	53 (25.9%)	25 (21.7%)	0.498
Thyroid disease, n (%)	27 (13.2%)	15 (13.2%)	1.000
Cardiac disease, n (%)	18 (8.8%)	12 (10.4%)	0.690
Sleep disorder, n (%)	16 (7.8%)	16 (13.9%)	0.085
Parkinson disease, n (%)	21 (10.2%)	8 (7.0%)	0.418
Other neurologic disease, n (%)	27 (13.2%)	14 (12.2%)	0.863
Pulmonary disease, n (%)	10 (4.9%)	6 (5.2%)	1.000

MMP-9 = matrix metalloproteinase-9.

#### Comparisons between the qualitative results of the MMP-9 immunoassay and symptoms of dry eye

There were no statistically significant differences in OSDI and VAS between the MMP-9positive and MMP-9negative groups (p > 0.05; Table 2).

**Table 2:** Comparison of clinical signs and symptoms in MMP-9 positive versus negative patients.

Parameters	MMP-9 positive (n = 205)	MMP-9 negative (n = 115)	*P* value
OSDI score, mean (SD)	35.2 (23.7)	33.8 (24)	0.628
VAS scale, mean (SD)	3.1 (2.7)	2.6 (2.8)	0.106
Schirmer score, mm, mean (SD)	4.7 (2.4)	4.7 (2.4)	0.919
tBUT, sec, mean (SD)	3.9 (2.6)	4.8 (3.6)	0.020*
tBUT, ≤3, n (%)	123 (60.0%)	56 (48.7%)	0.047*
Corneal staining, score, mean (SD)	1.2 (1.1)	0.9 (1.0)	0.012*
Corneal staining, ≥2, n (%)	94 (45.9%)	37 (32.2%)	0.018*
Conjunctival staining, score, mean (SD)	1.6 (2.0)	1.2 (1.8)	0.058
Conjunctival staining, ≥2, n (%)	94 (45.9%)	35 (30.7%)	0.008*
Meibomian gland turbidity, grade, mean (SD)	1.1 (0.7)	1.2 (0.6)	0.912
Meibomian gland expression, grade, mean (SD)	1.3 (1.1)	1.2 (1.0)	0.362
Meibomian gland dysfunction, ≥3, n (%)	108 (52.7%)	53 (46.1%)	0.411

MMP-9, matrix metalloproteinase-9; OSDI, ocular surface disease index; VAS, visual analogue scale; tBUT, tear break-up time.

*Statistically significant by independent two-sample t-test or Pearson’s chi-square test.

#### Tear volume measurements by tear meniscometry

There was no statistically significant difference in mean tear volume, as measured by tear meniscometry, between the MMP-9positive and MMP-9negative groups (p > 0.05; Table 2).

#### Tear breakup time

The mean tBUT of the MMP-9–positive group was shorter than that of the MMP-9–negative group, and it was statistically significant (p = 0.020). Furthermore, when stratified by tBUT <3 seconds, the MMP-9positive group showed significantly more patients with tBUT <3 seconds than the MMP-9–negative group (p = 0.047; Table 2).

#### Corneal and conjunctival stain score

The mean corneal stain score of the MMP-9–positive group was higher, and it was statistically significant (p = 0.012). When the analysis was stratified using a corneal stain score cutoff of > 2, significantly greater number of patients were included in the MMP-9 positive group than in the MMP-9 negative group (p = 0.018; Table 2). In addition, the mean conjunctival stain scores of the MMP-9positive and MMP-9negative groups were not significantly different (p > 0.05). When stratified by a conjunctival staining score >2, significantly more patients were observed in the MMP-9–positive group than that in the MMP-9–negative group (p = 0.008; Table 2).

#### MGD

The mean meibomian gland turbidity in the MMP-9–negative group was slightly higher than that of the MMP-9–positive group, but the difference was not statistically significant (p > 0.05). The grades of meibomian gland expression were not significantly different between the two groups (p > 0.05). A higher percentage of MGD grade >3 was noted in the MMP-9 positive group, but it was not statistically significant (p > 0.05; Table 2).

### Semiquantitative and quantitative results of the MMP-9 immunoassay

#### Demographics of the study populations

The demographics of the allocated patients are shown in Table 3. No difference in age, sex, systemic autoimmune disease, Sjögren syndrome, and systemic diseases (diabetes, hypertension, thyroid disease, cardiac disease, sleep disorder, Parkinson disease, other neurologic disease, and pulmonary disease) was observed in the four groups (p>0.05).

**Table 3:** Demographics of semiquantitative results of MMP-9 point-of-care test.

Parameters	Grade 0 (n = 115)	Grade 1 (n = 124)	Grade 2 (n = 50)	Grade 3 (n = 31)	*P* value
Age, mean (SD)	58 (13)	57 (13)	61 (12)	60 (16)	0.846
Gender, n (%) male	32 (28%)	32 (26%)	19 (38%)	14 (45%)	0.104
Systemic autoimmune disease (except Sjögren syndrome), n (%)	14 (12%)	14 (11%)	5 (10%)	1 (3%)	0.541
Sjögren syndrome, n (%)	9 (8%)	14 (11%)	10 (20%)	2 (6.5%)	0.111
Diabetes, n (%)	5 (4%)	9 (7%)	7 (14%)	3 (10%)	0.178
Hypertension, n (%)	25 (22%)	25 (20%)	17 (34%)	11 (36%)	0.102
Thyroid disease, n (%)	15 (13%)	17 (14%)	6 (12%)	4 (13%)	0.991
Cardiac disease, n (%)	12 (10%)	11 (9%)	3 (6%)	4 (13%)	0.726
Sleep disorder, n (%)	16 (14%)	8 (7%)	7 (14%)	1 (3%)	0.104
Parkinson disease, n (%)	8 (7%)	11 (9%)	6 (12%)	4 (13%)	0.636
Other neurologic disease, n (%)	14 (12%)	16 (13%)	5 (10%)	6 (20%)	0.663
Pulmonary disease, n (%)	6 (5%)	4 (3%)	4 (8%)	2 (7%)	0.590

MMP-9 = matrix metalloproteinase-9.

#### Comparisons between the semiquantitative results of the MMP-9 immunoassay and DE severity

No significant difference in age, OSDI, VAS, Schirmer score, tBUT, tBUT ≤3 seconds, corneal staining score, conjunctival staining score, conjunctival staining score ≥2, meibomian gland turbidity, expression, and dysfunction was observed in the four groups (p>0.05). However, significant difference was observed in corneal staining score ≥2 (p = 0.036; Table 4).

**Table 4:** Comparison of clinical signs and symptoms in semiquantitative results of MMP-9 point-of-care test.

Parameters	Grade 0 (n = 115)	Grade 1 (n = 124)	Grade 2 (n = 50)	Grade 3 (n = 31)	*P* value
OSDI score, mean (SD)	33.8 (24.0)	36.0 (24.1)	34.7 (24.7)	33.0 (20.9)	0.888
VAS scale, mean (SD)	2.5 (2.8)	3.2 (2.6)	3.0 (3.0)	2.6 (2.2)	0.288
Schirmer score, mm, mean (SD)	4.7 (2.4)	4.8 (2.5)	4.5 (2.5)	4.7 (2.4)	0.846
tBUT, sec, mean (SD)	4.8 (3.6)	3.7 (2.3)	4.2 (3.2)	4.0 (2.9)	0.060
tBUT, ≤ 3, n (%)	56 (48.7)	78 (63.0)	28 (56.0)	18 (58.1)	0.252
Corneal staining, score, mean (SD)	0.9 (1.0)	1.2 (1.1)	1.4 (1.1)	1.1 (1.0)	0.102
Corneal staining, ≥2, n (%)	29 (25.2)	49 (39.5)	23 (46.0)	11 (35.5)	0.036*
Conjunctival staining, score, mean (SD)	1.2 (1.8)	1.8 (2.1)	1.5 (1.8)	1.2 (1.5)	0.288
Conjunctival staining, ≥2, n (%)	37 (32.2)	63 (50.8)	20 (40.0)	11 (35.5)	0.457
Meibomian gland turbidity, grade, mean (SD)	1.2 (0.7)	1.2 (0.7)	0.9 (0.6)	1.2 (0.8)	0.074
Meibomian gland expression, grade, mean (SD)	1.2 (1.0)	1.3 (1.1)	1.3 (1.1)	1.5 (1.2)	0.660
Meibomian gland dysfunction, ≥3, n (%)	53 (46.1)	65 (52.4)	24 (48.0)	18 (58.1)	0.388

MMP-9, matrix metalloproteinase-9; OSDI, ocular surface disease index; VAS, visual analogue scale; tBUT, tear break-up time.

*Statistically significant by the linear-by-linear association chi-square test.

#### Clinically significant associations with semiquantitative MMP-9 immunoassay results

The tBUT showed significant negative correlation with the semiquantitative MMP-9 immunoassay results (*rho* = -0.125, p = 0.025; Table 5). In contrast, the corneal staining score showed significant positive correlation with the semiquantitative MMP-9 immunoassay results (*rho* = 0.122, p = 0.029; Table 5). Other parameters, such as age, OSDI score, VAS score, Schirmer score, MGD, and conjunctival staining score, showed nonsignificant correlation (p > 0.05; Table 5).

**Table 5:** Clinically significant associations with semiquantitative results of MMP-9 point-of-care test.

Parameters	*rho* value	*P* value
Age	0.094	0.093
OSDI score	0.012	0.842
VAS score	0.074	0.199
tBUT	-0.125	0.025*
Schirmer score	-0.017	0.766
Meibomian gland dysfunction	0.033	0.613
Conjunctival staining score	0.060	0.281
Corneal staining score	0.122	0.029*

MMP-9, matrix metalloproteinase-9; OSDI, ocular surface disease index; VAS, visual analogue scale; tBUT, tear break-up time.

*Statistically significant by the linear Spearman’s rank correlation test.

#### Clinically significant associations with quantitative MMP-9 immunoassay results

The age, OSDI, VAS, Schirmer score, tBUT, corneal staining score, conjunctival staining score, and MGD showed clinically nonsignificant correlation with the quantitative MMP-9 immunoassay results (p > 0.05; Table 6).

**Table 6:** Clinically significant associations with quantitative results of MMP-9 point-of-care test.

Parameters	*r* value	*P* value
Age	0.021	0.720
OSDI score	0.037	0.539
VAS score	-0.009	0.276
tBUT	-0.015	0.293
Schirmer score	-0.019	0.748
Meibomian gland dysfunction	0.034	0.622
Conjunctival staining score	0.021	0.720
Corneal staining score	0.113	0.052

MMP-9, matrix metalloproteinase-9; OSDI, ocular surface disease index; VAS, visual analogue scale; tBUT, tear break-up time.

## Discussion

DE is a chronic condition affecting 5% to 30% of the population aged 50 years or older [1]. Traditionally, symptoms of DE are evaluated using DE questionnaires, and DE signs are assessed by tBUT, corneal staining score, conjunctival staining score, tear film assessment, and the Schirmer test [1, 26]. However, the pathogenesis of DE is still not fully understood. During the past 20 years, clinicians have paid more attention to inflammation and recognized its key role in the development and amplification of signs and symptoms of DE [27]. MMP-9 is a nonspecific biomarker of inflammation, and elevated tear MMP-9 levels were found in DE [14, 17]. Aragona et al. [28] demonstrated that MMP-9 levels measured by polymerase chain reaction correlated well with corneal or conjunctival stain scores and tBUT. Quantitative analysis of MMP-9 using enzyme-linked immunosorbent assay had been introduced; however, this method is time-consuming and expensive, rendering it difficult to use routinely in clinics [29]. To overcome these drawbacks, the MMP-9 immunoassay (InflammaDry) was developed. This immunoassay has advantages of low cost, rapid results, and ease of device preparation [30].

Although InflammaDry is a point-of-care immunoassay developed to discriminate between DE and non-DE, clinicians inevitably became curious about the clinical meaning of weak positive and strong positive results. In addition, the basic principle of InflammaDry is that the band is expressed through a colorimetric reaction; thus, there is a possibility that the distinctness of the band may vary in proportion to the concentration of MMP-9, that is, the degree of clinical ocular surface inflammation.

In the present study, we evaluated the result of tear MMP-9 immunoassay test using qualitative, semiquantitative, and quantitative analyses of immunoassay band. In the qualitative analysis, shorter tBUT, tBUT ≤3 seconds, higher corneal staining score, corneal staining score ≥2, and conjunctival staining score ≥2 were related to a positive MMP-9 immunoassay result. This implies that positive MMP-9 immunoassay results are correlated with severity of clinical signs of DE. The instability of the tear film in DE induces tear hyperosmolarity, which subsequently induces inflammatory reactions on the corneal epithelium. These inflammatory cascades on the ocular surface result in the release of inflammatory cytokines. Therefore, the positivity of the MMP-9 immunoassay could be related to the severity of clinical signs of DE [31, 32]. Covita et al. [33] reported that more severe MGD was associated with a shorter tBUT. However, no differences in MGD were observed between the MMP-9 positive and negative groups in this study. These discrepancies may be because MGD was subjectively assessed by the clinicians in our study, and not by imaging using near-infrared illumination. In the semi-quantitative analysis, we found that shorter tBUT and higher corneal stain score, which already showed significant differences in the qualitative analysis, were correlated to the semiquantitative MMP-9 immunoassay results. Shimazaki-Den et al. [34] showed the close relation of mucin components with tBUT. As mucin plays an important role in maintaining corneal health and is secreted by the goblet cells, and diminished goblet cell density is observed in chronic inflammation such as allergy [35]. Similarly, severe ocular surface inflammation in DE decreases mucin secretion, resulting in a shorter tBUT or higher corneal staining score. Thus, a highly reactive band was observed using the MMP-9 immunoassay. This finding is also consistent with that of Park et al. [19], to some extent, who demonstrated good correlation between semiquantitative MMP-9 grading and DE signs and symptoms. In our results, however, we could not identify any correlation between clinical symptoms and MMP-9 positivity. This is because the symptoms of DE are nonspecific, as similar symptoms can present in various ocular surface diseases, and Pflugfelder et al. [36] reported that only 57% of symptomatic patients showed clinical signs of DE.

Interestingly, the quantitative analysis using ImageJ, which is considered most accurate way to evaluate the tear MMP-9 levels, showed no correlation between the immunoassay band density and the clinical signs and symptoms of DE. This finding is quite contrary to the above mentioned as the semiquantitative analysis of MMP-9 immunoassay was related to the clinical severity of DE. The reason for the discrepancies between the semiquantitative and quantitative results might be that the semiquantitative results are only based on the interpretation of reagent band density, whereas the quantitative results are based on both the reagent and control band densities. Same reagent band density can be read differently in the semiquantitative and quantitative analyses of MMP-9 immunoassay. Our previous studies showed that the band density of the MMP-9 immunoassay reagent is influenced by tear volume and thus, it could not accurately indicate MMP-9 concentration in tears [15, 20]. However, the previous validation of the immunoassay was conducted under experimental conditions, and the control band density was not considered. Therefore, the quantitative analysis used in this study to assess individual tear volume might be more indicative of the correlation between the quantitative test results and clinical signs of DE, and could be used in future studies. From these, positive MMP-9 immunoassay patients tend to show severe ocular signs of DE; however, interpretation of quantitative measures of reagent band densities for assessing clinical severity of DE need further studies with considering the individual tear volume.

The following limitation must be considered: First, the possibility of selection bias must be considered, as the study was conducted in a tertiary hospital. Patients with uncontrolled evaporative DE in primary or secondary hospitals are likely to be referred to our hospital and thus, included in this study. The subtypes of DE were not assessed in this study. Therefore, further studies of MMP-9 positivity in aqueous tear-deficient DE and evaporative DE are needed. Second, we did not evaluate the tear MMP-9 levels in this study. A recent study reported that an increase in band density was observed as the MMP-9 concentration increased [37]. However, measurement of the tear MMP-9 levels and analysis of the semi-quantitative and quantitative results of the MMP-9 immunoassay could provide more accurate information. Finally, follow-up comparisons of clinical signs and symptoms of DE with successive MMP-9 immunoassays were not performed in this study. Soifer et al. [38] reported that MMP-9 positivity could predict long-term decrease in tear production, and anti-inflammatory therapy to decrease MMP-9 concentration to undetectable levels may be beneficial in such cases. Further long-term follow-up studies of semi-quantitative or quantitative analyses of MMP-9 immunoassays to assess the efficacy of anti-inflammatory therapy would help establish treatment guidelines for DE patients.

In conclusion, the positive MMP-9 immunoassay results are related to the severity of ocular signs of DE. However, using quantitative indicators of MMP-9 immunoassay for assessing clinical severity of DE requires further investigation.

## Supporting information

S1 Dataset (XLSX)

S1 Fig Graphical results. Association of tear matrix metalloproteinase 9 immunoassay with signs and symptoms of dry eye disease.
(TIF)
