# Supplementary figures and images for: Association of tear matrix metalloproteinase 9 immunoassay with signs and symptoms of dry eye disease: A cross-sectional study using qualitative, semiquantitative, and quantitative strategies
Source: PLoS One. 2021 Oct 18;16(10):e0258203. doi: 10.1371/journal.pone.0258203 (PMC8523049; doi:10.1371/journal.pone.0258203)

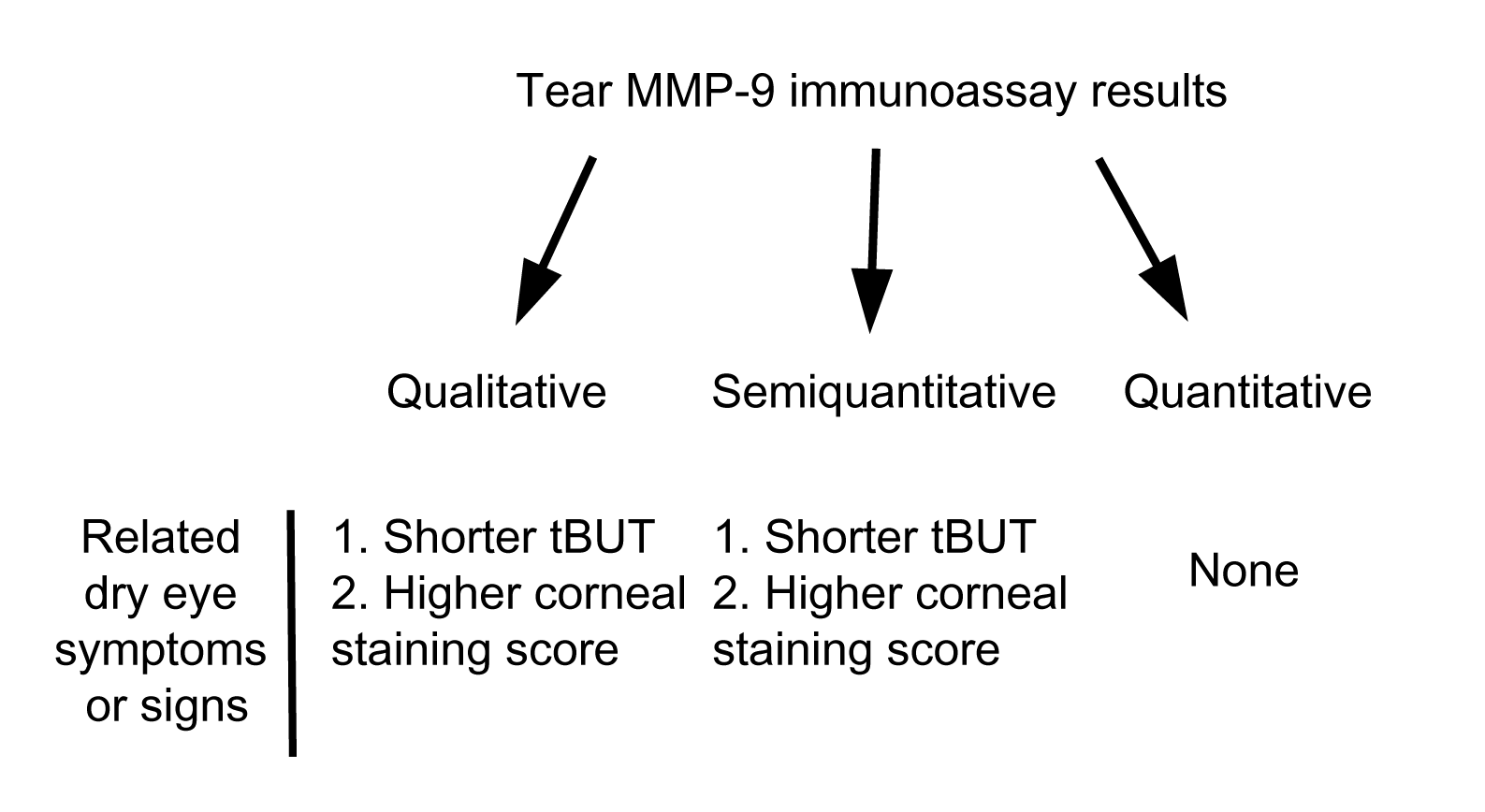

Supplement: S1 Fig — Association of tear matrix metalloproteinase 9 immunoassay with signs and symptoms of dry eye disease. (TIF) [file pone.0258203.s002.tif]
